# First Identification of CPV-2c Infection in a Wild Cub Giant Panda (*Ailuropoda melanoleuca*) Suggesting an Emerging Transmission From Wildlife and Domestic Dogs

**DOI:** 10.1155/tbed/6716483

**Published:** 2025-05-20

**Authors:** Ziyao Zhou, Xiaogang Shi, Kerong Li, Qiang Hu, Yuxin Ren, Xiaoxiao Zhou, Min Li, Ting Zhang, Fang Yang, Yuyan Huang, Chengdong Wang, Desheng Li, Zhijun Zhong, Haifeng Liu, Caiwu Li, Tingmei He, Guangneng Peng

**Affiliations:** ^1^Department of Clinical Veterinary Medicine, College of Veterinary Medicine, Sichuan Agricultural University, Chengdu, Sichuan, China; ^2^Sichuan Wolong National Natural Reserve Administration Bureau, Wenchuan, Sichuan, China; ^3^Chengdu Center for Animal Disease Prevention and Control, Chengdu, Sichuan, China; ^4^China Conservation and Research Center for the Giant Panda, Chengdu, Sichuan, China

**Keywords:** CPV-2c, the giant panda, VP2, wildlife, Wolong Nature Reserve

## Abstract

Canine parvovirus type 2 (CPV-2) is a member of the Parvoviridae family that causes several animals for diarrhea, vomiting, and even death, particularly in cubs. Previous evidence has shown that CPV-2 is capable of infecting giant pandas, causing mild intestinal symptoms. In November 2020, a dead young giant panda was discovered in the Wolong Nature Reserve in Sichuan, China. Through physical examination, anatomical pathology, histopathology, and PCR testing, the panda was diagnosed with CPV-2 infection. Further investigations into the CPV-2 epidemic among wildlife in the Wolong Nature Reserve revealed an epidemic situation with a 14.52% (9/62) positive rate in fecal samples of wild animals near 350 National Highway. In total, 40 canine fecal samples from the Wolong and nearby cities were next analyzed. Interestingly, all dog fecal samples from Wolong tested CPV-2 negative, while seven positive samples were successfully amplified from the urban samples. Partial VP2 gene analysis identified four giant panda strains and nine canine strains as CPV-2c variations, with shared nucleotide and amino acid homologies of 99.2%–100%, respectively. Phylogenetic analysis revealed that the CPV-2c strains in our study belonged to the same cluster of Chinese and Asian CPV-2c strains while distinct from European and American strains. This study is the first identification indicating that CPV-2c has significantly threatened the health and survival of wild cub giant pandas, which might originate from domestic dogs from near cities.

## 1. Introduction

Canine parvovirus type 2 (CPV-2) is an important virus that threatens muti-carnivores' health, which may cause vomiting, diarrhea, malabsorption of nutrients, translocation of intestinal bacteria, and leukopenia [1]. A previous study illustrated that the giant panda (*Ailuropoda melanoleuca*) could be infected by CPV-2, causing gastrointestinal symptoms [2]. The CPV-2 positive rate might be up to 50% in captive wild animals in zoos in China [2–4]. The genome of CPV is ~5.12 kb, with two crucial open reading frames (ORFs) [5] encoding nonstructural proteins (NS1 and NS2) and structural proteins (VP1 and VP2), respectively [6]. VP2 is the main capsid protein as well as an important antigenic protein [7]. According to the variation of the VP2 gene, CPV-2 is divided into three antigenic variants: CPV-2a, CPV-2b, and CPV-2c [8]. Various articles illustrated that different CPV-2 subtypes may contribute to the preference of infected animals and regions. Their virulence may also vary.

Wolong Nature Reserve is the home of wildlife in Sichuan, China. According to the records, the reserve harbors 149 wild giant pandas, which represent ~8% of the total wild panda population in China. Thanks to the concerted efforts of the government in nature conservation, the reserve currently supports various wild species, including tufted deer, golden snub-nosed monkeys, sambar deer, long-tailed gorals, takins, serows, etc. It is important to note that these animals can serve as natural reservoirs of CPV-2 and possess the potential to transmit the virus to giant pandas. Within the Wolong Nature Reserve, the Shenshuping Base of the China Conservation and Research Center for Giant Pandas (CCRCGP), is also housing dozens of captive giant pandas.

In November 2020, a dead young wild giant panda was discovered during a routine field inspection in the Wolong Nature Reserve. In this study, the cause of death of the giant panda was investigated, and further investigation was conducted into the prevalence of CPV-2 in the Wolong Township and surrounding cities.

## 2. Materials and Methods

### 2.1. Ethics Statement

The autopsy of the corpse and collection of wild animal samples were performed in compliance with the animal protection law of the People's Republic of China. This study was approved by the Wolong Nature Reserve Management Administration Bureau.

### 2.2. Autopsy and Histopathology of the Wild Cub Giant Panda

The dead giant panda cub was discovered in November 2020. Initially, a general assessment for age and gender was conducted by an experienced panda breeder. Next, basic measurements of the panda's body were taken. An autopsy was next performed under sterile conditions at the laboratory of Shenshuping Base, CCRCGP.

During the autopsy, tissue samples were collected from the heart, liver, spleen, lungs, kidneys, intestine, mesenteric lymph nodes, thymus, and bladder. These samples were preserved in 10% neutral-buffered formalin. Hematoxylin–eosin (HE) staining was applied to the samples. Microscopic analysis was performed to observe any histopathological changes.

### 2.3. Investigation of Intestinal Infectious Pathogens in the Wild Cub Giant Panda

Since the histopathological findings suggested intestinal inflammation in the deceased cub wild giant panda, further examinations by PCR and Colloidal gold rapid test strips (Shanghai Ronghui Biotechnology Co., Ltd.) for common gastrointestinal pathogens in giant pandas, including CPV-2, rotavirus (RV), canine distemper virus (CDV), canine adenovirus (CAV), and canine coronavirus (CCV) were conducted.

Intestinal tissue and fecal samples from the dead giant panda were collected and centrifuged at 8000 rpm for 2 min. DNA extraction was performed using a TIANamp Virus DNA/RNA Kit (Tiangen Biotech, Bejing Co., Ltd.) The primers are listed in Table 1. The colloidal gold test was conducted following the instructions in the manual. The PCR reaction conditions were as follows: initial denaturation at 95°C for 5 min, followed by 30 cycles of denaturation at 95°C for 30 s, annealing at 55°C for 30 s, extension at 72°C for 30 s; and a final extension at 72°C for 10 min.

### 2.4. Genotyping Analysis of Partial VP2 Gene for CPV-2

The CPV-2-positive samples were performed genotype detection using VP2 genotyping primers (VP2-2F and VP2-2R, as listed in Table 2). These primers were used to amplify a partial VP2 gene sequence, specifically a 706 bp segment located from position 622 to 1327 of the complete VP2 genome. The amplified fragments were then subjected to sequencing analysis [9]. The PCR reaction conditions were as follows: initial denaturation at 94°C for 3 min, followed by 40 cycles of denaturation at 94°C for 30 s, annealing at 50°C for 45 s, extension at 72°C for 1 min, and a final extension at 72°C for 7 min. The sequences were next splicing and trimming using DNAstar, resulting in 706 bp nucleotide sequences and translated into amino acid sequences. Antigenic variants were confirmed based on the presence of specific mutations at amino acid positions 297 and 426 in the VP2 protein [10, 11].

### 2.5. Epidemiological Samples

**Figure 1:**
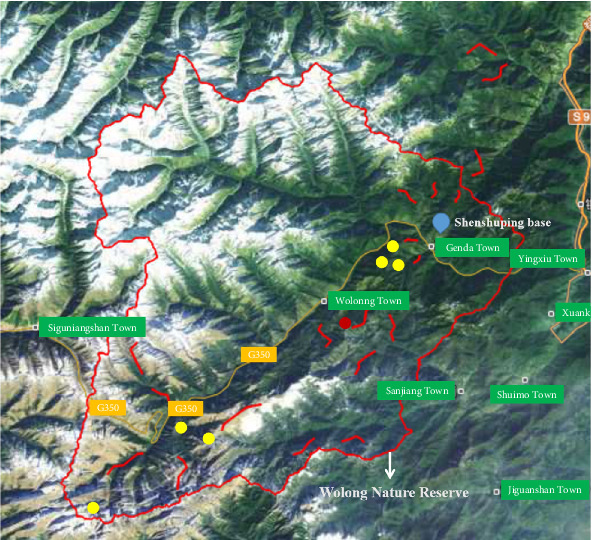
The distribution of sample collection routes and CPV-positive samples. Red lines were sample collection routes. Circle represents the positions of positive samples. Red circle refers to the place where the dead young giant panda was found and with two positive fecal samples. Along the yellow line is the 350 national highway. The figure was authorized by the Wolong Nature Reserve Administration.

Following the initial discovery of giant pandas infected with CPV-2, a large-scale survey was urgently conducted in Wolong Nature Reserve. The routes for sample collection are illustrated in Figure 1. Since dogs are the most common natural hosts of CPV-2, 20 fecal samples were also collected from dogs with diarrhea in Wolong and Gengda towns, respectively, as well as 20 suspected positive samples from veterinary hospitals in Chengdu and Ya'an cities, respectively, which are the closest cities to Wolong Nature Reserve. According to the method in Section 2.3, the fecal samples of wild animals and dogs were tested for CPV-2.

### 2.6. Genetic and Amino Acid Evolutionary Analysis of CPV-2

Sequenced positive samples were aligned with 51 reference sequences of CPV-2 (including seven sequences were from our laboratory collected in Chengdu City previously [12]) and FPLV (used as an outgroup), with different countries, hosts, and subtypes, from the NCBI database. Based on the 706 bp VP2 gene sequences, phylogenetic trees were constructed using the neighbor-joining method and maximum-likelihood method (1000 bootstrap) in MEGA. Amino acid sequences were also translated for the evolutionary analysis of CPV-2.

## 3. Results

### 3.1. Physical Examination and Autopsy

**Figure 2:**
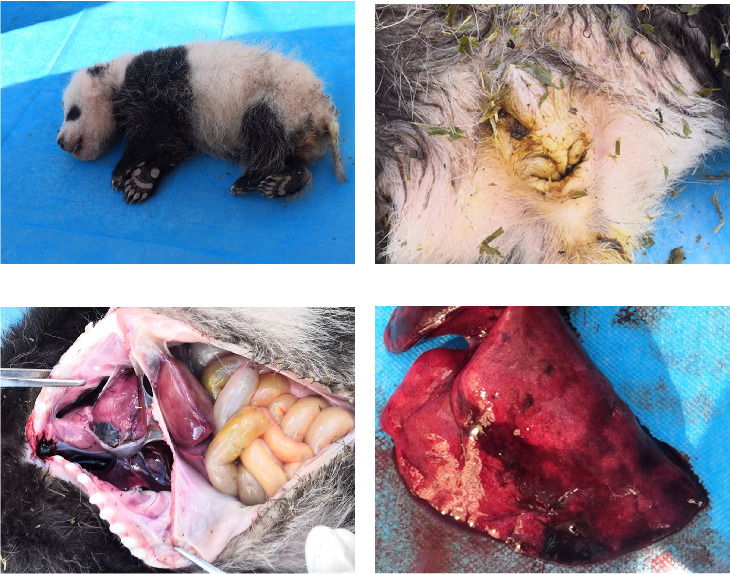
The dead panda cub and its anatomical pathology of it. (A) The overall condition of the young giant panda's corpse. (B) Loose stools at the anus and messy coat around the anus. (C) Organs in the thoracic cavity and abdominal cavity. Intestinal tissue was pale yellow with bleeding spots on the serosal surface. Liver was dark red with partial pressure marks. (D) The right lung was dark red with hemorrhage.

The wild cub giant panda was male, weighing 1.215 kg and measuring 26.00 cm in body length. Its age was estimated to be approximately 1 month old by an experienced giant panda breeder. During a general examination, the giant panda appeared thin and showed signs of diarrhea, as indicated by loose stools (Figure 2A,B). The mouth and nasal cavity were clean and free of foreign matter. The coat appeared normal, but subcutaneous congestion was observed on the right abdomen.

As per the autopsy (Figure 2C), the intestinal tissue showed a pale yellow color with bleeding spots on the serosal surface. A significant amount of gas was present inside. The liver displayed a dark red coloration with partial pressure marks. The right lung exhibited a dark red hue with signs of hemorrhage, while the left lung appeared normal (Figure 2D). No visible changes were observed in the other organs and tissues.

### 3.2. Histopathology Diagnosis

**Figure 3:**
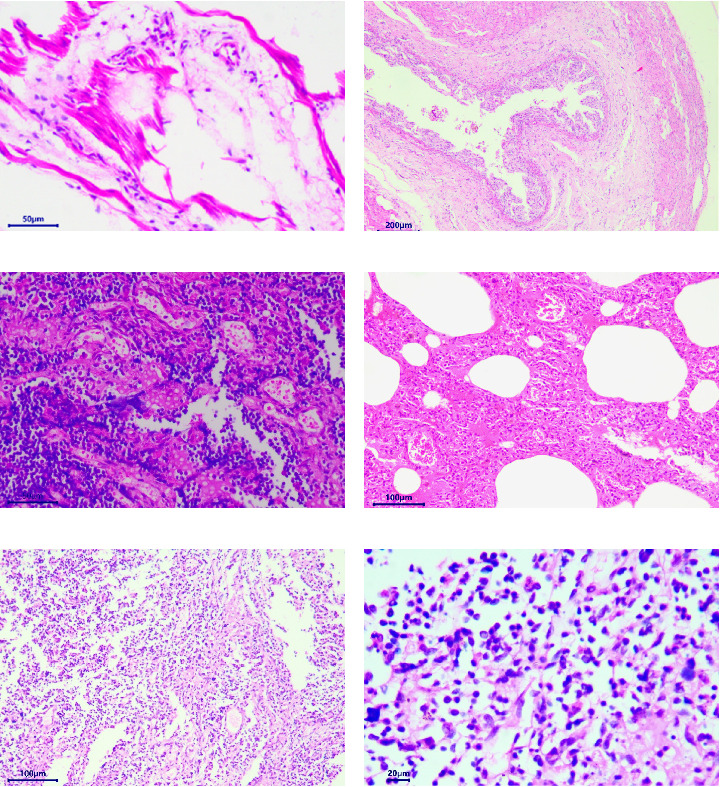
Histopathological picture (HE staining). (A) The muscle bundles of the myocardium were broken and dissolved, muscle filament spacing was widened, and edema was observed. A small number of macrophages and neutrophils infiltrated the myocardium. (B) Losses, inactivation, and fusions of villus. (C) Small blood vessels and capillaries of the thymus were congested, with obvious microthrombi. A few macrophages and neutrophils were seen in the thymus. (D) Inflammatory cell infiltrations were seen in the bronchioles but not in the larger bronchi. Alveolar walls were thickened without obvious inflammatory infiltration. The capillaries of the lung were congested, and the alveolar cavity was obviously dilated. (E) and (F) The structure of mesenteric lymph node was collapsed with a large number of neutrophils infiltrated.

The pathological examination revealed several indications. For example, the muscle bundles of the myocardium were broken and dissolved, with widened muscle filament spacing and observed edema. Some macrophages and neutrophils infiltrated the myocardium, suggesting the possibility of myocarditis (Figure 3A). The structures of the intestinal mucosa were destroyed due to the loss, inactivation, and fusion of villi (Figure 3B). Small blood vessels and capillaries in the thymus were congested, with evident microthrombi (Figure 3C). Inflammatory cell infiltrations were observed in the bronchioles but not in the larger bronchi (Figure 3D). The structure of the mesenteric lymph node was collapsed, with a large number of neutrophils infiltrating, indicating the occurrence of acute purulent inflammation (Figure 3E,F). Alveolar walls were thickened without obvious inflammatory infiltration. The capillaries of the lungs were congested, and the alveolar cavity was noticeably dilated. Renal tubules showed obvious protein casts, and the renal capsules were dilated. A few macrophages and neutrophils were observed in the thymus. The structure of the spleen was also disrupted, with unclear splenic white pulp.

### 3.3. Molecular Biology Diagnosis

Both the colloidal gold and PCR tests yielded the following results: positive for CPV and negative for RV, CDV, CAV, and CCV. The PCR product of CPV-2 was ~340 bp, consistent with expected size. The sequence was consistent with CPV-2 strains, confirming that CPV-2 existed in the intestinal tissue of the young giant panda.

### 3.4. Epidemiologic CPV-2 Investigation

After the discovery of the dead giant panda, urgent fecal samples were collected from the Wolong Nature Reserve. In this collection, a total of eight samples were obtained from giant pandas, 13 samples from snow leopards, and 41 samples from other wildlife species. Due to limitations in identifying the specific animal sources in the field, there were also four samples of unidentified animal feces (Table S1). Among them, nine samples were CPV-2 positive (positive rate was 14.06%). Positive samples came from four species: 50% of giant panda samples (4/8), 75% of leopard cat samples (3/4), 100% of hog badger (1/1), and 25% of unknown animals (1/4). The locations (latitude, longitude, and altitude) of positive samples are in Table 3. Notable, the distributions of all nine positive samples were not far from 350 national highways (Figure 1). Besides, the great majority of positive samples were also not far from Shenshuping Base; the nearest one of them was less than 6 km. What is more, the positive samples of giant pandas clustered together and closed to Niutou Mountain (Figure 1), heralding the occurrence of concentrated infections.

Fecal samples from diarrheal dogs in Genda and Wolong towns were also collected. However, all of the 20 diarrheal samples tested negative for CPV-2. Interestingly, we found there were seven positives among the urban samples. Among them, there were four positives in Ya'an and three positives in Chengdu.

### 3.5. Amplicon of Partial VP2 for Positive Samples

Amplification of the partial VP2 gene in the positive samples was further conducted to investigate their phylogenetic analysis. All four samples from the giant pandas were successfully amplified. Additionally, seven positive dog samples were also amplified successfully. However, we were unable to success amplify the partial VP2 gene from other wildlife species.

A total of 19 partial VP2 gene sequences were analyzed together, including four sequences were obtained from giant pandas (named WL20201101, WL20201102, WL20201103, and WL20201104), 11 sequences from dogs, including seven sequences were collected in our study (CDD3, CDD4, CDD10, YAD6, YAD9, YAD11, and YAD16) and four sequences were from our laboratory previously (CD202201, CD202102, CD202101, and CD202202). As a result, the four strains from giant pandas (WL20201101–WL20201104) had 100% identity. The nucleotide and amino acid homologies of these four giant panda CPV-2 sequences to 15 canine CPV-2 sequences were 99.2%–100% and 98.7%–100%, respectively. The sequences amplified in this study (besides four sequences from our laboratory previously) were submitted to NCBI Genbank with accession numbers PP862230–PP862244.

### 3.6. Amino Acid Mutation and Phylogenetic Analysis

Amino acid mutations of partial VP2 proteins among the four strains from the giant pandas and seven strains from the dogs were listed in Table 4. The taxonomical criteria of different antigenic variants, nine strains (four from the giant pandas, three from the dogs presented in Ya'an, and two from dogs presented in Chengdu) were identified as CPV-2c, while two were identified as new CPV-2a (one from dogs in Ya'an and one from dogs in Chengdu). All 11 strains had the mutations Phe267Tyr and Tyr324Ile. Mutation Gln370Arg was also found in all CPV-2c strains in our study, which was considered unique in CPV-2c. All two new CPV-2a strains had the Thr440Ala substitution. Besides, there was a unique CPV-2c strain (CDD4) that also appeared in the substitution of Thr440Ala, which made the strain in special.

**Figure 4:**
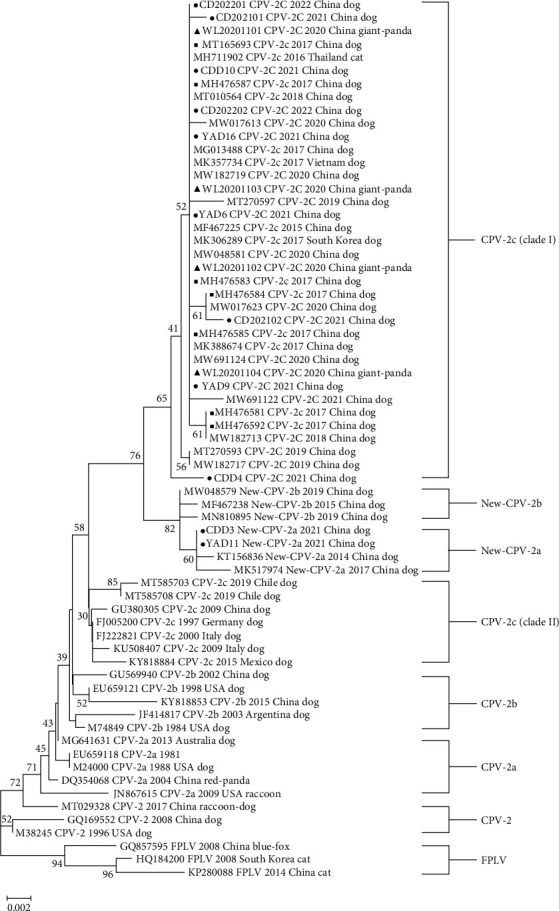
Neighbor-Joining phylogenetic tree based on partial VP2 gene sequences. Triangles (▲) represent the CPV strains from giant panda. Circles (●) were the canine CPV strains from our study and our laboratory. Squares (■) were the reference CPV-2C strains from Chengdu. Feline panleukopenia virus (FPLV) was used as an outgroup reference. Values on branches next to nodes represent bootstrap analysis results. Reference strains were labeled as accession number, subtypes, collection year, country, and host. The strains in this study were labeled as strain's name, subtypes, collection year, country, and host.

The phylogenetic tree based on the partial VP2 gene is shown in Figure 4 (NJ) and Figure S1 (ML), respectively. All CPV-2c strains formed two clusters (Clade I and Clade Ⅱ). Clade I includes Chinese CPV-2c strains after the year 2015 and strains from other Asian countries such as Thailand, South Korea, and Vietnam. Clade Ⅱ consists of early Chinese CPV-2c strains and other strains from Europe and America, including Italy, Mexico, and Germany. The CPV-2c strains analyzed in our study clustered with cluster I while distant to Clade Ⅱ. The giant panda strains (WL20201101-04) were most similar with CPV-SH1516 and MG013488, which is the representative strain of CPV-2c in China. The two new CPV-2a strains in our study (CDD3 and YAD11) clustered along with the new CPV-2a reference strains while distant to the CPV-2a cluster.

## 4. Discussion

CPV-2 has emerged as a significant threat to the health and even survival of mammals. Clinical observations indicate that CPV-2 primarily affects young animals and often leads to fatal outcomes in puppies [13]. During the infection process, the virus initially invades the oropharyngeal lymph nodes, mesenteric lymph nodes, and thymus and subsequently spreads to the intestinal epithelial crypts, bone marrow, tongue epithelium, oral cavity, cardiac myocytes, lungs, spleen, liver, and kidneys [14]. Without treatment, CPV-infected dogs may have a mortality rate of up to 91% [15, 16].

The presence of loose stools and intestinal lesions in the giant panda suggested gastrointestinal disorders, which are the most common clinical symptoms of CPV-2 infection. The pressure marks on the liver might be attributed to the accumulation of gas in the intestine. Gastrointestinal disorders can lead to the translocation of intestinal bacteria into the bloodstream, resulting in septic shock, multiorgan failure, systemic inflammatory response syndrome, and potentially death if left untreated [14]. Additionally, inflammatory cell infiltrations in the mesenteric lymph nodes, bronchioles, thymus, and myocardium indicated the occurrence of systemic inflammation in the giant panda. The presence of inflammatory infiltrations in the bronchioles but not in the larger bronchi further supported the notion that the inflammatory infection might originate from the bloodstream rather than an external source. Furthermore, myocarditis was suspected in the cub panda due to the evident pathological changes and inflammatory infiltrations observed in the myocardium. Myocarditis is known to be a common cause of death in CPV-infected animals [17]. Finally, the giant panda also exhibited signs of multiple organ dysfunction syndrome and blood coagulation disorders. Affected organs include the lungs, heart, kidneys, gastrointestinal tract, spleen, thymus, and lymph nodes. The gross physical examination, anatomical lesions, and histopathological changes observed in the giant panda align with the characteristics of CPV-2 infection described in dogs and other animals [18–22]. However, since the panda we found has been dead for several days, the pathology may not be accurate enough for the direct death evidence. Only the PCR detection results confirmed the presence of CPV-2 infection in the giant panda.

Previously, the CPV-2 positive rate of serosurvey was ~50% in zoos in Sichuan and Chongqing, China [2]. In 1994, six of eight giant pandas were detected as CPV antibody-positive in Wolong, China [3]. Later, in 2010, Qin et al. [4] performed a serosurvey of 67 giant pandas from Wolong and found that 48% of the unvaccinated giant pandas were CPV antibody-positive. Additionally, Guo et al. [23], in 2013, detected the CPV-2 virus in the giant panda feces. A canine review has also revealed that CPV-2 was widely distributed in China, with 36% prevalence in dogs, while much higher in Sichuan province (58%) [24]. Similar to dogs, three serosurvey researches also showed high infection rates (~50%) of CPV-2 in giant pandas [2–4]. The positive rate of CPV-2 in the giant pandas in our study was 44.4% that is similar to that of previous studies.

CPV-2 has a wide range of hosts; in addition to domestic cats and dogs, it can also be detected in wolf, fox, Siberian tiger, red panda, giant panda, Taiwanese pangolin, crab-eating fox, raccoon, civet cat, serval, and other wild carnivores [20, 25–32]. Wolong belongs to high-altitude regions with low temperatures; thus, viruses in samples would not be eliminated quickly. According to epidemiological investigations in our study, leopard cat and hog badger suffered from CPV-2 in Wolong Nature Reserve. Our results showed that the positive rate of CPV2 in badger was 100%. This result has some limitations due to the challenges and uncertainties associated with wildlife sampling; there was only one sample of badger, which has potential implications for the investigation of CPV2 positive rate in wild animals. Meanwhile, due to the challenges of collecting fecal samples in the wild and the subsequent handling process, in this case, the partial VP2 gene was failed to amplify for further genetic evolutionary analysis despite best efforts.

Previous evidence has indicated that CPV-2 can be widespread between wildlife and domestic dogs [33–35]. Interest, in our study, all positive samples in Wolong wildlife were along the 350 national highway. Many travelers with pet dogs from the city would pass by or temporarily live along the 350 national highway, which creates an opportunity for CPV-2 transferring from the city to Wolong Nature Reserve. Therefore, diarrhea canine fecal samples in close towns and cities were further collected and detected. The similar partial VP2 genetic relationship of wild giant panda and domestic dog CPV-2 were further confirmed the across transmission risk.

Based on the amino acid residues 426 and 296, four CPV-2 strains from the giant panda and nine canine strains were identified as CPV-2c, while another two canine strains were identified as new CPV-2a. Reference mutation Phe267Tyr may be associated with the transmission and infection of virus other than antigenicity [36]. Residue 323 plays an important role when virus binding to the host transferrin receptor (TfR) [37]. Mutation Tyr324Ile adjacent to residue 323 may strong this binding and affect the host range [11]. Gln370Arg was also observed in all CPV-2c strains but not in other variants consistent with other studies. Mutation Gln370Arg can enlarge the host range and is connected with hemagglutinating function [38]. The two new CPV-2a strains in our study showed a substitution of Thr440Ala, which has been reported in new CPV-2a and new CPV-2b in China [11, 16]. Notably, this mutation was also found in a CPV-2c strain (CDD4) in our study that is not reported in Chinese CPV-2c strains but in Chilean CPV-2c strains [39]. From the phylogenetic tree, CDD4 belonging to Clade I did not cluster with Chilean CPV-2c strains (MT585708 and MT585703), suggested that this mutation was likely from local adaption, not derive from the introduction of other countries. Due to the limitations and uncertainties of many samples in wild animals, the discussion of virulence can only be used as a reference but not a definitive virulence result.

CPV-2c first appeared in 2000 year, Italy, later than CPV-2a and CPV-2b [40]. In China, CPV-2c was first reported in 2010 [41] and was increasingly detected in the following years [42]. It is a remarkable fact that CPV-2c variants have existed in Chengdu since 2016 and had a relatively large proportion [11]. Recently, more and more CPV etiological surveys in China showed increased prevalence rates of CPV-2c, which had exceeded CPV-2a. For example, in Guangdong, the components of CPV-2c and CPV-2a isolates were 39.47% (15/38) and 36.84% (14/38), respectively [43]; Chen et al. [16] revealed that the proportions of CPV-2c and new CPV-2a isolates from different provinces in China were 70% (21/30) and 23% (7/30), respectively; Hu et al. [44] also found that CPV-2c strains had a 80.33% proportion during 2018–2019 among three provinces; Jiang et al. [45] found CPV-2c was 70.4% in all 44 isolates from 2018 to 2019 in Jilin Province.

Interest, CPV-2c did not initially hold a dominant position in China. Hao et al. [43] analyzed 1076 CPV-2 sequences from China collected over various periods. The results of their analysis revealed that CPV-2a was the predominant strain before 2015, reaching its peak prevalence in 2014 before showing a decline. However, from 2017 to 2018, CPV-2c emerged and replaced CPV-2a as the dominant variant in China. This finding was further supported by Chen et al. [16], who analyzed 683 Chinese CPV strains collected between 2014 and 2019. The dominance of CPV-2c over CPV-2a was observed not only in China but also in other Asian countries, Vietnam [46, 47], Laos [47], Korea [48], Mongolia [49], and Thailand [49]. These findings suggest that CPV-2c has a high transmission capacity and has been increasingly prevalent in the region.

As far as we know, our study is the first detection of CPV-2c from the giant panda. Several reports have indicated that CPV exists in giant pandas and can result in diseases, but most of the reports were serosurvey not focusing on CPV antigenic variants [2–4, 23, 50]. Previously, Guo et al. [23] identified a new CPV-2a strain (CPV-2a OVC) from giant panda. However, the CPV strain in giant pandas of our study was CPV-2c, quite different from strain CPV-2a OVC. A reasonable explanation might be as follows: the sample of strain CPV-2a OVC was collected from 2011 to 2012 when CPV-2a predominated; strains from giant pandas in our study were identified as CPV-2c probably related to the rapid epidemic of CPV-2c in China.

The phylogenetic analysis in our study revealed that the four giant panda strains were clustered together with the nine canine CPV-2c strains, as well as CPV-2c strains from Chengdu in 2017. Previous phylogenetic analyses have shown that most CPV-2c isolates from China formed a monophyletic cluster, except for the earliest strains in 2009 [16, 42]. The phylogenetic tree based on the partial VP2 gene illustrated that CPV-2c strains formed two clusters. The CPV-2c strains analyzed in our study clustered with Clade I (later Asian) while distant to Clade Ⅱ (European, American, and earlier Asian). This suggests that the later Chinese CPV-2c strains likely originated from a mutation of the original CPV rather than being introduced from European or American countries. The CPV-2c strains detected in the giant pandas were likely transmitted from the later Chinese CPV-2c strains and were closely related to the dominant strains of CPV-2c found in Ya'an and Chengdu.

Our epidemic generated a strong risk of CPV transmission to captive giant pandas in the Shenshuping base, as the nearest position of the positive sample was only 6 km away from the base. Viruses might transform from the infected animals through feces, vomit, and saliva, contaminating food and water for susceptible animals. Since the virus was able to survive for a long time in vitro in the low temperature in Wolong Nature Reserve, preventing CPV-2 transmission became challenging [14, 51]. Research shows vaccination can cross-protect against CPV-2a and CPV-2c in dogs [52]. Therefore, emerging vaccination for local domestic dogs was performed under the supervision of the local administration bureau.

In conclusion, the epidemic situation of CPV in Wolong Nature Reserve was severe in 2020, causing the death of young giant panda and infections of multiple wildlife. The viral variant of giant pandas was CPV-2c with Phe267Tyr, Tyr324Ile, and Gln370Arg mutations in the VP2 protein. The prevalence of CPV in Wolong Nature Reserve may come from the transmission of canine CPV-2c from nearby cities.

## Figures and Tables

**Table 1:** Common enterovirus detection primers for giant pandas.

Virus	Gene	Primer	Size (bp)
RV	VP7	5′-GTATGGTATTGAATATACCAC-3′	342
		5′-GATCCTGTCGGCCATCC-3′	

CDV	N	5′-CG-GAAATCAACGGACCTAAAT-3′	242
		5′-TCCTTGAGCTTTCGACCCTT-3′	

CPV	VP2	5′-CCATGGTACAGATCCAGATGA-3′	342
		5′-GCCTCAAAAGAATAATATGGT-3′	

CCV	S	5′-CACACATTCTGATGGAGACG-3′	372
		5′-AAACCTCCTAGCCAAGAACC-3′	

CAV	F	5′-TGGTTGGGAGCTTTCTCACTC-3′	1,019
		5′- AGGGGATGAACTCGGCTGT-3′	

**Table 2:** The primers of CPV-2 partial VP2 gene.

Gene	Primer name	Primer sequence (5′-3′)	Size (bp)
VP2	VP2-2F	5′-TGG AGATAT TAT TTTCAATGGGAT A-3′	706
	VP2-2R	5′-TAATTCCTGYTTTACCTCCAA-3′	

**Table 3:** The location of CPV-positive samples.

Animal species	Latitude	Longitude	Altitude
Giant panda (dead)	103.21337	31.00571	2817
Giant panda	103.21337	31.00571	2817
Giant panda	103.21337	31.00571	2817
Giant panda	103.25597	31.07762	2694
Leopard cat	103.01375	30.88081	3168
Leopard cat	103.27069	31.09514	2388
Leopard cat	103.27627	31.07552	2074
Hog badger	103.04241	30.86123	4025
Unknown animal	102.91657	30.78601	3796

**Table 4:** Amino acid mutations in partial VP2 protein of CPV strains from giant pandas and dogs.

Accession number	Strain name	Variants	Host	Country	Collection date	267	297	300	305	324	370	426	440
M38245	CPV-b	CPV-2	Dog	USA	1996	F	S	A	D	Y	Q	N	T
EU659118	CPV-13.us.81	CPV-2a	Dog	USA	1981	—	—	G	Y	—	—	—	—
M24000	CPV-31	CPV-2a	Dog	USA	1988	—	—	G	Y	—	—	—	—
JF414817	Arg5	CPV-2b	Dog	Argentina	2003	—	N	G	Y	—	—	D	—
M74849	39	CPV-2b	Dog	USA	1984	—	—	G	Y	—	—	D	—
KT156836	HRB-F8	New-CPV-2a	Dog	China	2014	Y	A	G	Y	I	—	—	A
MK517974	CN/HN1709	New-CPV-2a	Dog	China	2017	Y	A	G	Y	I	—	—	A
MF467238	CPV-HN1520	New-CPV-2b	Dog	China	2015	Y	A	G	Y	I	—	D	A
MN810895	CC-38	New-CPV-2b	Dog	China	2019	Y	A	G	Y	I	—	D	A
KU508407	CPV_IZSSI_25835_09	CPV-2c	Dog	Italy	2009	—	A	G	Y	—	—	E	—
FJ222821	56/00	CPV-2c	Dog	Italy	2000	—	A	G	Y	—	—	E	—
MG013488	CPV-SH1516	CPV-2c	Dog	China	2017	Y	A	G	Y	I	R	E	—
Our study	CDD10	CPV-2c	Dog	Chengdu, China	2021	Y	A	G	Y	I	R	E	—
Our study	CDD4	CPV-2c	Dog	Chengdu, China	2021	Y	A	G	Y	I	R	E	A
Our study	YAA6	CPV-2c	Dog	Yaan, China	2021	Y	A	G	Y	I	R	E	—
Our study	YAA9	CPV-2c	Dog	Yaan, China	2021	Y	A	G	Y	I	R	E	—
Our study	YAA16	CPV-2c	Dog	Yaan, China	2021	Y	A	G	Y	I	R	E	—
Our study	WL20201101	CPV-2c	Giant panda	China	2020	Y	A	G	Y	I	R	E	—
Our study	WL20201102	CPV-2c	Giant panda	China	2020	Y	A	G	Y	I	R	E	—
Our study	WL20201103	CPV-2c	Giant panda	China	2020	Y	A	G	Y	I	R	E	—
Our study	WL20201104	CPV-2c	Giant panda	China	2020	Y	A	G	Y	I	R	E	—
Our study	CD202101	CPV-2c	Dog	Chengdu, China	2021	Y	A	G	Y	I	R	E	—
Our study	CD202102	CPV-2c	Dog	Chengdu, China	2021	Y	A	G	Y	I	R	E	—
Our study	CD202201	CPV-2c	Dog	Chengdu, China	2022	Y	A	G	Y	I	R	E	—
Our study	CD202202	CPV-2c	Dog	Chengdu, China	2022	Y	A	G	Y	I	R	E	—
Our study	CDD3	New-CPV-2a	Dog	Chengdu, China	2021	Y	A	G	Y	I	—	—	A
Our study	YAA11	New-CPV-2a	Dog	Yaan, China	2021	Y	A	G	Y	I	—	—	A

## Data Availability

The sequencing data are available in the NCBI database with the accession numbers PP862230–PP862244. Maximum-Likelihood phylogenetic tree based on partial VP2 gene sequences and the number of the samples of animal species were listed as Supporting Information.

## Supporting Information

Supporting Information Figure S1. Maximum-likelihood phylogenetic tree based on partial VP2 gene sequences. Table S1. Animal species and the number of the samples.
