# Supplementary material for: First Identification of CPV-2c Infection in a Wild Cub Giant Panda (Ailuropoda melanoleuca) Suggesting an Emerging Transmission From Wildlife and Domestic Dogs
Source: Transbound Emerg Dis. 2025 May 20;2025:6716483. doi: 10.1155/tbed/6716483 (PMC12116211; doi:10.1155/tbed/6716483)
Supplement: Supporting Information — Figure S1. Maximum-likelihood phylogenetic tree based on partial VP2 gene sequences. Table S1. Animal species and the number of the samples. [file 6716483.f1.docx]

Supplementary


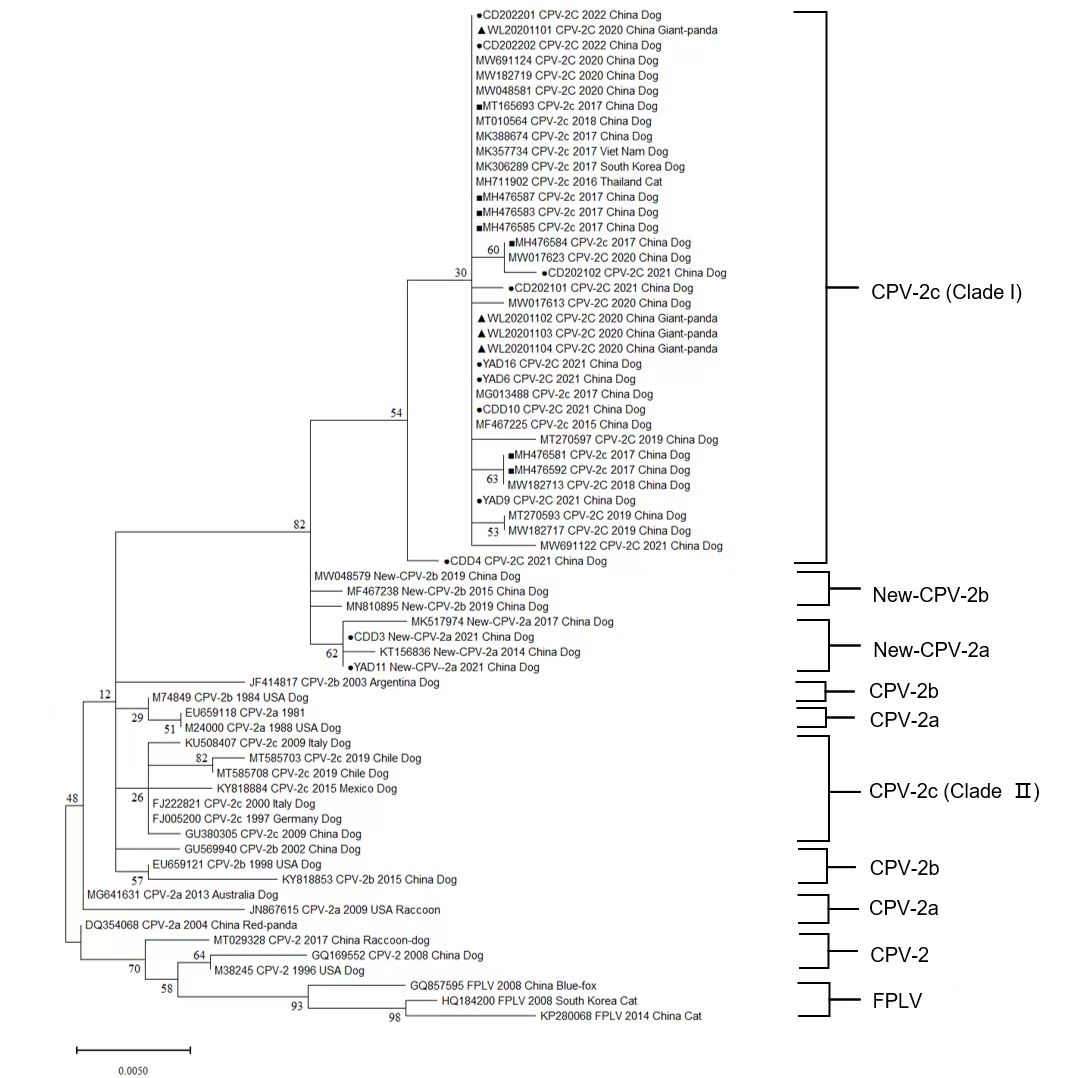


Figure S1 Maximum-Likelihood phylogenetic tree based on partial VP2 gene sequences. Triangles (▲) represent the CPV strains from giant panda. Circles (●) were the canine CPV strains from our study and our laboratory. Square (■) were the reference CPV-2C strains from Chengdu. Feline panleukopenia virus (FPLV) is used as an outgroup. Values on branches next to nodes represent bootstrap analysis results. Reference strains are labelled as accession number, subtypes, collection year, country, and host. The strain in this study is labelled as strain's name, subtypes, collection year, country, and host.

Table S1 Animal species and the number of the samples

| Animal species | Number of samples | Animal species | Number of samples |
| --- | --- | --- | --- |
| Giant panda | 8 | Stone marten | 1 |
| Snow leopard | 13 | Masked civet | 1 |
| Sambar deer | 8 | Black bear | 1 |
| Blue sheep | 8 | Blood pheasant | 1 |
| Leopard cat | 4 | Antelope | 1 |
| Chinese monal | 2 | Porcupine | 1 |
| Musk deer | 2 | Serow | 1 |
| Long-tailed goral | 2 | Takin | 1 |
| Tufted deer | 2 | Unknown animal | 4 |
| Hog badger | 1 | Total | 62 |
